# The Relation between Occupational Sitting and Mental, Cardiometabolic, and Musculoskeletal Health over a Period of 15 Years – The Doetinchem Cohort Study

**DOI:** 10.1371/journal.pone.0146639

**Published:** 2016-01-11

**Authors:** H. Susan J. Picavet, L. Willemijn Pas, Sandra H. van Oostrom, Hidde P. van der Ploeg, W. M. Monique Verschuren, Karin I. Proper

**Affiliations:** 1 Centre for Nutrition, Prevention and Health Services, National Institute for Public Health and the Environment, Bilthoven, The Netherlands; 2 Department of Public and Occupational Health, EMGO+ Institute for Health and Care Research, VU University Medical Center, Amsterdam, The Netherlands; CSIR-Indian Institute of Toxicology Research, INDIA

## Abstract

**Objective:**

Sedentary behaviors are reported to impose health risks. Since occupational exposure is a major proportion of total sedentary time, we studied the association between occupational sitting and a number of health problems.

**Methods:**

From the longitudinal Doetinchem Cohort Study, we selected those working at baseline with complete data (n = 1,509). Participants were examined four times at 5 year-intervals between 1993 and 2012. We characterized occupational sitting as follows: 1) stable sitters and stable non sitters over a 15-year period, based on job characteristics and (2) having a job with a low, moderate or high amount of sitting, based on tertiles of self-reported number of hours per week of occupational sitting, measured at wave 5. Linear and logistic regression models were used. Outcomes were self-reported mental health, low-back or upper extremity pain, and objectively measured cardiometabolic health (overweight, hypertension, hypercholesterolemia).

**Results:**

Compared to stable non sitters, a lower risk of chronic upper extremity pain was observed for stable sitters (OR 0.75, 95% CI: 0.57; 1.00) as well as for those in the two upper tertiles for hours of occupational sitting (>4 hr/wk) (OR 0.65; 95%CI 0.50–0.86). For the other health outcomes studied, no significant associations were found with occupational sitting.

**Conclusion:**

Our findings do not support the hypothesis that occupational sitting is associated with health problems. The finding that occupational sitting is associated with less upper extremity pain might be due to the association of occupational sitting with less physical load.

## Introduction

Sedentary behavior is different from physical inactivity and refers to any waking behavior characterized by an energy expenditure ≤1.5 while in a sitting or reclining posture [1]. Examples of sedentary behaviors are sitting, lying down, watching TV, and other forms of screen-based behaviors. In the past decade, research on sedentary behaviors as a health threat has increased exponentially and has shown that it may be associated with an increased risk of major health outcomes, including all-cause and cardiovascular mortality [2–4], cardiovascular disease, type II diabetes [5,6], and cancer [2,7–9].

Most of the research to date has focused on either total sitting time or on leisure-time sitting, particularly on TV viewing or other screen-based behaviors. Although increasing, still little attention has been paid to other domains where people sit a lot, such as in the workplace.

Due to increasing technological developments, work has become less physically demanding and more mentally demanding, thereby often being more sedentary [10]. Consequently, the majority of the workforce sits most of the day. In the Netherlands, workers sit on average about 7 hours per day (on working and non-working days), of which one third was at work [11]. Also, Australian data showed that working adults spent up to one half of the workday sitting down implying occupational sitting is a main contributor to total daily sitting time [12,13]. In a systematic review, Van Uffelen et al. (2010) found limited evidence for a detrimental association between occupational sitting and different health outcomes [9]. Their conclusions were hampered by heterogeneity between studies and lack of high quality studies.

As the majority of the adults is working, and thereby sedentary during a large part of the day, it is important to get more insight into the potential adverse health impact of occupational sitting. Especially knowledge on the long-term detrimental health effects of occupational sitting is unknown due to the lack of studies. The aim of the present study is to exploit existing long-term data for the question on the relation between occupational sitting and different health outcomes. The focus was on the relation between long-term occupational sitting with mental health, cardiometabolic health, and musculoskeletal pain, using data over a period of 15 years.

## Methods

### Study participants

Data was used from the Doetinchem Cohort Study. At baseline (1987–1992), each year, an age- and sex stratified random sample of 12,405 male and female inhabitants of Doetinchem between 20 and 60 years old was invited to participate. From the participants of this baseline examination, a random sample of 7,769 was invited to participate in a second examination (1993–1997), and again five, ten and fifteen years later for a third (1998–2002), fourth (2003–2007) and fifth examination (2008–2012). Participants who actively declined to participate, were not invited again.

All participants gave a written informed consent for participation in the study. The Medical Ethics Committee of the Netherlands Organization of Applied Scientific Research Institute approved the study. More details on data collection of the Doetinchem Cohort Study can be found elsewhere [14].

For the research question of this paper, data was used from the second round until the fifth round, covering 15 years of follow-up for individual participants. For the present analyses, only working participants were selected, based on a question whether they had paid work with seven answer categories: paid employment (payroll), self-employed, housewife/-man, unemployed, retired, disabled, other. Those working as an employee (payroll) and self-employed (n = 3,597 at baseline) were selected. At each new measurement round, there was a loss to follow-up of around 20%. In addition, there was exclusion due to missing values on the variables of interest. Data of 1,694 workers on occupational sitting and health outcomes were available across the four rounds. Analyses were also performed to the time spent sitting at work and health, both measured at round 5. Complete data on these main variables (at round 5) involved 1,967 workers.

### Variables of interest

#### Occupational sitting

Occupational sitting was assessed with two questions that refer to 1) sedentary work or 2) hours of occupational sitting. 1) From the second to the fifth round (1993–2012), respondents were asked about their occupational physical (in)activity as part of a questionnaire on physical activity.[15] With a single item question, respondents could indicate whether their job was mainly sedentary (such as: desk work), or mainly standing (such as: shop assistant, barber, horeca), manual (such as: nurse, plumber, electrician), or involved high physical loads (such as: construction worker, cleaner, farmer). 2) In the fifth round, respondents were also asked to report on the number of hours sitting at work during a usual week (in hours per week) over the past 12 months.

#### Mental health

Mental health was measured by means of the Mental Health Inventory (MHI-5), which includes five questions about feelings of nervousness and depression during the past four weeks and a six-point response scale [16]. A sum score was rescaled from 0 to 100, whereby a higher score indicated a better mental health. The MHI-5 has shown to be a reliable and valid measure of mental health status [16,17].

#### Cardiometabolic health

The following cardiometabolic risk factors were assessed and used in the present study: body mass index (BMI) and overweight, hypertension, and hypercholesterolemia.

*BMI* (in kg/m^2^) was calculated by body weight in kg divided by height in meters squared. Body weight and height were measured during a physical examination by trained staff. During measurement, participants wore light indoor clothing with emptied pockets and no shoes. Body weight was assessed on a SECA balance scale to the nearest 100 g. Height was measured with a wall-mounted stadiometer to an accuracy of 0.5 cm. Overweight (including obese) was defined as having a BMI ≥25 kg/m [18].

*Blood pressure* was measured twice by a trained professional while the participant was in sitting position. The mean of the two measurements was taken. Blood pressure was measured with a Speidel–Keller meter (Welch Allyn, Skaneateles Falls, NY, USA) with the first-phase Korotkoff and fifth-phase Korotkoff sound as criteria for systolic and diastolic blood pressure, respectively. Hypertension was defined as a systolic blood pressure of ≥140 mmHg and/or a diastolic blood pressure of ≥90 mmHg.

*Total cholesterol* was measured in serum, using standardized enzymatic methods. Hypercholesterolemia was defined as ≥6.5 mmol/l.

#### Musculoskeletal health

Musculoskeletal health was defined as the 12-month prevalence as well as chronic pain in the lower back and in the upper extremities. Self-reported 12-month prevalence of pain in the *lower back* was measured using the question ‘have you had any trouble, discomfort or pain in the lower back during the last 12 months?’ (yes, no) [19]. Chronic low back pain was determined by a subsequent question on duration and defined as pain ≥3 months [20].

For the 12-month prevalence of *upper extremity* pain, participants were asked to report their 12-month prevalence (yes/no) of trouble, discomfort or pain in the high back, shoulder, neck, elbow, and wrist or hand using five sub-questions per upper body region. If a person indicated having pain in one of the above mentioned body regions, that person was identified as having pain in the *upper extremities*. Chronic upper extremity pain was, similar to low back pain, defined as a duration of ≥3 months.

#### Covariates

Potential confounders in the association between sitting and health outcomes, measured at baseline (i.e. second round), were included in the analyses. In addition to gender and age, the highest educational level achieved was assessed and divided into three categories: low (intermediate secondary education or less), moderate (intermediate vocational or higher secondary education), and high (higher vocational education or university). Marital status was defined as married or not. Respondents were also asked to indicate their working hours per week in a usual week in the last 12 months. Smoking of cigarettes was dichotomized into ‘current smoker’ or ‘non/ex-smoker’. Leisure time physical activity (PA) was measured with a validated self-administered questionnaire [15]. This questionnaire includes time spent on PA during work, leisure time, household activities, transportation, sports and other strenuous activities. Adherence to PA guidelines was defined as meeting the goal of being physically active at a moderate or vigorous level for at least 30 minutes a day for 5 or more days a week. The cut-off point for reaching this levels was set at 3.5 hours a week spent on at least moderately intense physical activities to account for the observation that the amount of PA is usually over-reported [20]. Sitting during leisure time was recorded at round 5 with a similar questionnaire format as used for sitting at work. Participants were asked to report their weekly sitting time during leisure time in a usual week over the past 12 months while: a) reading and/or studying, b) watching TV, c) sitting behind the computer, and d) other sitting activities (talking with friends, playing games, listening to music, etc.).

### Statistical analysis

We used two different strategies to characterize occupational sitting. Using the data over 15 years, workers were divided into stable sitters or stable non sitters based on the characterization of their job. Stable sitters were defined as workers who had indicated to have a sedentary job at least three out of the four measurements, whereas stable non sitters were defined as those having indicated at least three out of four times not having a sedentary job. Participants who indicated to have switched twice between a sedentary job and non-sedentary job, or who switched in the last round were excluded from this particular part of the present analysis (n = 185) to study the maximal contrasting two groups. Thus, the study population used for the analyses with respect to stable sitting jobs was 1,509 (1,694–185).

Further, as weekly hours spent of occupational sitting was only measured at round 5, workers were divided cross-sectionally into having a job with a low (0–3 hours/week), moderate (4–20 hours/week) or high amount of sitting (>20 hours/week) based on tertiles.

Descriptive characteristics were calculated for the study population stratified by occupational sitting status using the longitudinal data (i.e. stable sitters versus stable non sitters).

The association between occupational sitting and the health outcomes was examined using linear and logistic regression models. Separate analyses were performed for each health outcome, measured at round 5. Three different models were used: model 1was adjusted for age, gender, and education; model 2 was additionally adjusted for working hours a week, smoking, meeting the PA recommendations, and leisure time sitting; model 3 was additionally adjusted for all other health outcomes, since these are all considered as potential confounders. All statistical analyses were performed using SAS software version 9.3 (Statistical Analysis System; SAS Institute, Inc., Cary, North Carolina).

## Results

### Characteristics study population

Table 1 shows the characteristics of the study population, measured at round 5, divided into stable sitters at work (n = 745) and stable non sitters at work (n = 764). The stable sitters were predominantly men (62%) and higher educated (42%), which differed from stable non sitters (54% and 20%, respectively, *p*<0.001). A large difference was observed in hours sitting at work between stable sitters and stable non sitters (26 versus 4 hours/week, *p*<0.001), but their leisure time sitting was similar (26 versus 24 hours/week). The study population had on average a good mental health (MHI-5 score 82 and 80) [21]. Further, 43% to 53% had upper extremity or low back pain in the past year, 60% and 59% were overweight, and about one third had hypertension (Table 1).

**Table 1:** Characteristics of the study population based on round 5 data (2008–2012) of the Doetinchem Cohort (n = 1,509).

	Stable sitters (n = 745)	Stable non sitters (n = 764)
Age (mean, SD)	54 (6)	53 (6)
Men (%)***	62	54
Educational level (% high)***		
• % low	16	42
• % moderate	42	38
• % high	42	20
Working hours (per week) (mean, SD)***	34 (11)	31 (13)
Sitting at work (hours/week) (mean, SD)***	26 (11)	4 (6)
Sedentary job (%)***	76 (27)	14 (18)
Smoking (% smoker)*	14	21
Adherence to PA guidelines (%)***	54	66
Sitting during leisure time* (hours/week) (mean, SD)	26 (12)	24 (11)
Mental health (score 0–100)	82 (13)	80 (14)
Overweight or obese (≥25 kg/m^2^) (%)	60	59
Hypertension (SBP/DP: ≥ 140/ ≥90 mmHg) (%)	36	34
Hypercholesterolemia (≥ 6.5 mmol/l) (%)	19	20
*12- month pain prevalence (%)*		
• Upper extremities	50	53
• Low back	44	43
*Chronic pain (%)*		
• Upper extremities **	22	29
• Low back	15	16

SD = standard deviation; PA = physical activity; SBP = systolic blood pressure; DBP = diastolic blood pressure

* *p*<0.05

** *p*<0.01

*** *p*<0.001 based on chi square of t-test for differences between stable sitters and stable non sitters

### Relation between stable occupational sitting over 15 years and health outcomes

Stable sitting over 15 years was not associated with mental health or cardiometabolic health outcomes (Table 2). For musculoskeletal health, stable sitters reported chronic pain less frequently in the upper extremities compared to stable non sitters (Model 3: OR = 0.75, 95%CI = 0.57, 1.00).

**Table 2:** Longitudinal association between stable occupational sitting^a^ and health outcomes^b^, based on Doetinchem Cohort study, round 2, 3, 4 and 5, 1993–2012.

		Model 1^c^ (N = 1,506–1,509)	Model 2^c^ (N = 1,374–1,377)	Model 3^c^ (N = 1,372)
Mental health	ß (CI)	0.71 (-0.72; 2.14)	0.59 (-0.92; 2.10)	0.51 (-0.99; 2.00)
Overweight (≥25 kg/m^2^)	OR (CI)	0.99 (0.79; 1.24)	0.94 (0.74; 1.19)	0.93 (0.73; 1.19)
BMI (kg/m^2^)	ß (CI)	-0.14 (-0.56; 0.28)	-0.25 (-0.68; 0.19)	-0.26 (-0.69; 0.17)
Hypertension^d^	OR (CI)	1.04 (0.82; 1.32)	1.08 (0.84; 1.39)	1.10 (0.86; 1.42)
Increased cholesterol^d^	OR (CI)	0.89 (0.67; 1.16)	0.80 (0.60; 1.07)	0.80 (0.60; 1.08)
*12-month pain prevalence*				
• Upper extremities	OR (CI)	0.98 (0.79; 1.22)	0.96 (0.76; 1.21)	0.97 (0.77; 1.23)
• Low back	OR (CI)	1.07 (0.86; 1.33)	1.06 (0.84; 1.34)	1.09 (0.86; 1.38)
*Chronic pain*				
• Upper extremities	OR (CI)	**0.76 (0.59; 0.98)**	**0.77 (0.59; 1.00)**	**0.75 (0.57; 1.00)**
• Low back	OR (CI)	1.05 (0.78; 1.41)	1.08 (0.78; 1.49)	1.17 (0.83; 1.65)

*Note*: Boldface indicates statistical significance (*p*<0.05).

^a^ Reference group = stable non sitters at work.

^b^ Health outcomes were measured at round 5.

^c^ Model 1: adjusted for age, gender, and education, measured in round 2; Model 2: additionally adjusted for working hours a week, smoking, complying with the PA guideline, and leisure time sitting; Model 3: full model, i.e. additionally adjusted for the remaining health outcomes

ß = beta, OR = odds ratio, CI = 95% confidence interval. A positive beta indicates a higher mental health score or BMI (kg/m^2^); an OR above 1 indicates more people reporting pain, having overweight, hypertension or increased cholesterol.

^d^ Hypertension defined as systolic blood pressure ≥ 140 and/or diastolic blood pressure ≥90 mmHg; Increased cholesterol defined as ≥ 6,5 mmol/l.

### Association between time spent on occupational sitting and health outcomes

There was no association between the time spent on occupational sitting and any of the health outcomes, except for chronic upper extremity pain (Table 3). Workers with a job with a moderate (4–20 h/week) or high (>20 h/week) number of hours sitting had a lower risk of chronic upper extremity pain compared to those with a job with few hours of sitting (0–3 h/week) (model 3: OR = 0.65, 95%CI = 0.50, 0.86, and OR = 0.65, 95%CI = 0.47, 0.89, respectively).

**Table 3:** Cross-sectional association between occupational sitting time (tertiles)^a^ and health outcomes, Doetinchem Cohort study, round 5, 2008–2012.

		Model 1^b^ (n = 1,967)		Model 2^b^ (n = 1,958)		Model 3^b^ (n = 1,950)	
		Occupational sitting					
		4–20 h/wk	>20 h/wk	4–20 h/wk	>20 h/wk	4–20 h/wk	>20 h/wk
Mental health	ß (CI)	0.25 (-1.29; 1.79)	0.91 (-0.74; 2.56)	0.69 (-0.93; 2.30)	0.64 (-1.14; 2.43)	0.48 (-1.12; 2.08)	0.45 (-1.31; 2.22)
Overweight (≥25 kg/m^2^)	OR (CI)	0.98 (0.77; 1.24)	0.93 (0.73; 1.20)	0.92 (0.72; 1.18)	0.84 (0.64; 1.10)	0.92 (0.71; 1.19)	0.82 (0.62; 1.09)
BMI (kg/m^2^)	ß (CI)	-0.02 (-0.46; 0.42)	0.02 (-0.46; 0.49)	-0.04 (-0.50; 0.41)	-0.19 (-0.69; 0.31)	-0.00 (-0.45; 0.44)	-0.16 (-0.66; 0.33)
Hypertension^c^	OR (CI)	1.00 (0.78; 1.27)	1.00 (0.77; 1.30)	0.95 (0.74; 1.24)	0.97 (0.73; 1.29)	1.00 (0.76; 1.30)	1.03 (0.77; 1.37)
Hypercholesterolemial^c^	OR (CI)	0.89 (0.67; 1.18)	1.00 (0.74; 1.35)	0.87 (0.64; 1.17)	0.92 (0.66; 1.28)	0.87 (0.64; 1.18)	0.93 (0.67; 1.29)
*12-month prevalence pain*							
• Upper extremities	OR (CI)	0.83 (0.66; 1.04)	0.95 (0.74; 1.21)	0.85 (0.67; 1.09)	0.96 (0.73; 1.25)	0.86 (0.67; 1.10)	0.86 (0.67; 1.10)
• Low back	OR (CI)	1.06 (0.84; 1.33)	1.08 (0.85; 1.38)	1.07 (0.84; 1.34)	1.11 (0.86; 1.42)	1.11 (0.88; 1.40)	1.16 (0.90; 1.50)
*Chronic pain*							
• Upper extremities	OR (CI)	**0.70 (0.55; 0.90)**	**0.69 (0.53; 0.91)**	**0.68 (0.52; 0.88)**	**0.67 (0.50; 0.90)**	**0.65 (0.50; 0.86)**	**0.65 (0.47; 0.89)**
• Low back	OR (CI)	1.10 (0.82; 1.48)	1.04 (0.75; 1.44)	1.10 (0.80; 1.51)	1.05 (0.73; 1.50)	1.25 (0.89; 1.75)	1.18 (0.81; 1.72)

*Note*: Boldface indicates statistical significance (*p*<0.05)

^a^ Occupational sitting time categories were based on tertiles of weekly hours of occupational sitting; low: 0–3 h/wk (reference group), moderate: 4–20 h/wk: high: >20 h/wk

^b^ Model 1: adjusted for age, gender, and education, measured at round 2; Model 2: additionally adjusted for working hours a week, smoking, complying with the PA guideline, and leisure time sitting; Model 3: full model, i.e. additionally adjusted for remaining health outcomes

ß = beta, OR = odds ratio, CI = 95% confidence interval. A positive beta indicates a higher mental score or BMI; an OR above 1 indicates more people reporting pain, having overweight, hypertension or increased cholesterol.

^c^ Hypertension defined as a systolic blood pressure ≥ 140 and/or a diastolic blood pressure ≥90 mmHg; Hypercholesterolemial defined as ≥ 6,5 mmol/l.

## Discussion

Data from our cohort study showed that workers with a sedentary job over a 15-year period did not show an increased risk for the studied health problems compared to workers with long-term non-sedentary jobs. In contrast, those stable sitters even seem to report chronic upper extremity pain less frequently than stable non sitters. Also for occupational sitting time, there were no associations with any of the health outcomes, except for chronic upper extremity pain. Workers who sit at their work for four or more hours per week were at decreased risk for chronic upper extremity pain than workers with low occupational sitting time (0–3 hours per week occupational sitting).

A systematic review from 2010 suggests that there is limited evidence to support a relation between occupational sitting and health risks, due to the low number of high quality studies, and heterogeneity between the studies identified [9]. Recent cross-sectional studies suggested that workers with mostly sitting jobs are at increased risk for overweight or obesity [22,23]. Chau and colleagues (2012) found a cross-sectional association between both leisure time and occupational sitting with obesity risk, although for occupational sitting the association was less distinct than for leisure time sitting [22]. The stronger association for leisure time sitting (than for occupational sitting) was also observed for cardiometabolic outcomes in other studies [24–26].

Our finding that occupational sitting is associated with less upper extremity pain might be due to the fact that sedentary jobs involve less physical workload. High physical load represents a risk factor for musculoskeletal pain [27–29].

Limited evidence is available on the association between occupational sitting and mental health. Based on earlier analyses in the Doetinchem Cohort Study, no association was found between occupational sitting and mental health, but an association was found between leisure time sitting, particularly watching TV and poorer mental health [30]. Studies on sitting behaviors and mental health have been more focused on non-occupational sitting (especially watching TV and other screen-based behaviors) and depression, and overall show a positive association between sedentary behaviors and depression [31]. A plausible explanation for the lack of association for occupational sitting with mental health may be related to the social withdrawal hypothesis [32]. Namely, as most jobs take place within a social context, workers will likely not feel lonely or depressed by a lack of social interaction. Another theory that has been suggested in the field of sedentary behavior is the ‘breaks theory’[33] suggesting that breaks in sitting time, like in occupational sitting, are beneficially associated with metabolic risk factors and thus explain the currently limited evidence for a health effect of occupational sitting. Moreover, in research to occupational sitting, the healthy worker effect may be present and yield selection bias from the overall healthy working population. In sum, while the relation between occupational sitting and health still needs further study of high quality, mechanisms underlying the eventual health effects of sitting are clearly complex and are an important area for further study.

Although some of the occupational sitting questionnaires have shown acceptable validity [34–37], for future research, it is recommended to use objective measurements such as accelerometers, preferably in combination with daily logs of working hours to also identify occupational and non-occupational sitting behavior [38].

Some limitations and strengths of this study need to be highlighted. First, as described above, self-reported occupational sitting is a limitation and may be a source of measurement error. Another methodological issue is that participants in cohort studies such as the Doetinchem Cohort Study have a relatively healthy profile. Non-respondents were more frequently overweight, lower educated, smokers, and inactive compared with complete cases [39]. However, for studying the relation between occupational sitting and health, eventual selective response is a minor issue. The data show large enough variation in the exposure and outcomes under study, making the analyses to the relation adequate. Furthermore, there is always the issue of possible residual confounding. A factor that may influence the relation between occupational sitting and health is type of job. We had only limited data on occupational history, and type of job and workload were not available in detail. Another aspect of socio-economic status is educational level, for which we did account for. However, effect sizes did not change considerably after adjustment implying no or minimal influence of educational level. Finally, for the data on the time spent sitting while at work, we were only able to use data of one round (fifth round, 2008–2012), and data were thus analyzed at a cross-sectional level, yielding no clues to the sequence of events.

The main strength of this study is the use of occupational sitting data over a long period, i.e. 15 years. Using the long-term prospective period, we were able to identify temporal patterns of occupational sitting, and distinguish between stable occupational sitters and stable non sitters. It should however be noted that we analyzed the health outcome at round 5, implying that we can draw a conclusion about the relation between occupational sitting and health at a later time, but not on the relation between occupational sitting and change in health. As this is one of the few studies on the health effects of occupational sitting over such a long period of time, we strongly recommend for more research on the long-term health effects of occupational sitting. Another strength is the focus on various health outcomes. Although the Doetinchem Cohort study involves a large population of more than 4,000 adults, we could only use a sample of the working population with complete data over the 15 year period. Still, with a study population of over 1,500 with complete data, there was sufficient statistical power to explore the relation between occupational sitting and health outcomes.

## Conclusion

We found no confirmation of the hypothesis that occupational sitting affects health negatively. Workers with stable occupational sitting appeared not to be at increased risk for mental problems, overweight, hypertension, hypercholesterolemia, or musculoskeletal health problems compared to workers with stable occupational non sitting. More research with longitudinal data and objective measurements of occupational sitting time are needed to confirm our findings and to strengthen the evidence about health risks due to sitting while at work.
